# Natural non-homologous recombination led to the emergence of a duplicated V3-NS5A region in HCV-1b strains associated with hepatocellular carcinoma

**DOI:** 10.1371/journal.pone.0174651

**Published:** 2017-04-10

**Authors:** Hélène Le Guillou-Guillemette, Adeline Pivert, Elise Bouthry, Cécile Henquell, Odile Petsaris, Alexandra Ducancelle, Pascal Veillon, Sophie Vallet, Sophie Alain, Vincent Thibault, Florence Abravanel, Arielle A. Rosenberg, Elisabeth André-Garnier, Jean-Baptiste Bour, Yazid Baazia, Pascale Trimoulet, Patrice André, Catherine Gaudy-Graffin, Dominique Bettinger, Sylvie Larrat, Anne Signori-Schmuck, Hénia Saoudin, Bruno Pozzetto, Gisèle Lagathu, Sophie Minjolle-Cha, Françoise Stoll-Keller, Jean-Michel Pawlotsky, Jacques Izopet, Christopher Payan, Françoise Lunel-Fabiani, Christophe Lemaire

**Affiliations:** 1 Laboratoire de Virologie, CHU Angers, France; 2 HIFIH Laboratory, UPRES 3859, SFR 4208, LUNAM University, Angers, France; 3 Laboratoire de Virologie, CHU Clermont-Ferrand, France; 4 Département de Bactériologie-Virologie-Hygiène Hospitalière et Parasitologie-Mycologie, CHRU, LUBEM, Brest, France; 5 Laboratoire de Virologie, CHU Limoges, France; 6 Laboratoire de Virologie, CHU Pitié-Salpêtrière, Paris, France; 7 Laboratoire de Virologie, CNR VHE, Inserm U1043, CHU Purpan, Toulouse, France; 8 AP-HP, GHU Cochin, Laboratoire de Virologie, Université Paris Descartes, Paris, France; 9 Laboratoire de Virologie, CHU Hôtel Dieu, Nantes, France; 10 Laboratoire de Virologie, CHU, Dijon, France; 11 Laboratoire de Virologie, CHU Avicenne, Bobigny, France; 12 Laboratoire de Virologie, Hôpital Pellegrin Tripode, CHU Bordeaux, France; 13 Laboratoire de Virologie, Centre de Biologie Nord, Hôpital de la Croix Rousse, Lyon, France; 14 Université François Rabelais, Inserm U966, CHU Tours, France; 15 Laboratoire de Virologie, CHU Besançon, France; 16 Laboratoire de Virologie, UMI 3265 UJF-EMBL-CNRS, CHU, Unit of Virus Host Cell Interactions, Grenoble, France; 17 Laboratoire de Bactériologie-Virologie, CHU Saint-Etienne, France; 18 Laboratoire de Virologie, CHU Rennes, France; 19 Institut de Virologie, CHU Strasbourg, Inserm U748, Strasbourg, France; 20 Laboratoire de Virologie-Bactériologie, CHU Henri-Mondor, Créteil, France; 21 IRHS, Université d’Angers, SFR4207 QUASAV, Angers, France; National and Kapodistrian University of Athens, GREECE

## Abstract

**Background:**

The emergence of new strains in RNA viruses is mainly due to mutations or intra and inter-genotype homologous recombination. Non-homologous recombinations may be deleterious and are rarely detected. In previous studies, we identified HCV-1b strains bearing two tandemly repeated V3 regions in the NS5A gene without ORF disruption. This polymorphism may be associated with an unfavorable course of liver disease and possibly involved in liver carcinogenesis. Here we aimed at characterizing the origin of these mutant strains and identifying the evolutionary mechanism on which the V3 duplication relies.

**Methods:**

Direct sequencing of the entire NS5A and E1 genes was performed on 27 mutant strains. Quasispecies analyses in consecutive samples were also performed by cloning and sequencing the NS5A gene for all mutant and wild strains. We analyzed the mutant and wild-type sequence polymorphisms using Bayesian methods to infer the evolutionary history of and the molecular mechanism leading to the duplication-like event.

**Results:**

Quasispecies were entirely composed of exclusively mutant or wild-type strains respectively. Mutant quasispecies were found to have been present since contamination and had persisted for at least 10 years. This V3 duplication-like event appears to have resulted from non-homologous recombination between HCV-1b wild-type strains around 100 years ago. The association between increased liver disease severity and these HCV-1b mutants may explain their persistence in chronically infected patients.

**Conclusions:**

These results emphasize the possible consequences of non-homologous recombination in the emergence and severity of new viral diseases.

## Introduction

Evolution by genome recombination has been widely described in numerous RNA viruses and shown to be relevant for epidemiology. To date, two models of RNA recombination have been described. The most frequently described and best-characterized model consists in a replicative mechanism with RNA template switching during viral RNA replication [1, 2]. The second model involves a non-replicative joining of RNA fragments [3]. Evolution by recombination is associated with both immune evasion and therapeutic resistance as illustrated by the emergence of drug resistant variants in HIV-infected patients receiving antiretroviral therapy [4]. Viral recombination may enhance viral pathogenicity, modify the host range or even contribute to the emergence of new viral strains, for example the Western equine encephalitis virus [5]. Also, recombination can have important consequences in immunization, for example the apparition of vaccine-derived poliovirus with regained virulence and contagiousness [6].

However, the frequency of recombination in the *Flaviviridae* family is low. Among the *Flaviviridae*, hepatitis C virus (HCV) is a major cause of liver disease. Recent estimates suggest that worldwide, at least 180 million people are positive for anti-HCV antibodies [7]. Chronically infected patients are at risk of developing liver cirrhosis and hepatocellular carcinoma, making HCV a major public health issue [8, 9]. Like other RNA viruses such as HIV, HCV is characterized by its wide genetic diversity: currently, seven genotypes and more than 67 subtypes are described [10]. Both of the recombination models discussed above have been described in HCV [11]. Also, rare, naturally recombinant strains have been identified at the inter- and intra-genotypic levels [12–15] with no change in gene length (i.e. no insertion); thus, none of these works described duplicated regions. However, HCV recombinations may be underestimated due to a lack of accurate, routine screening tools [16, 17]. A recent work using ultra-deep pyrosequencing to analyze HCV subpopulations during intra-host evolution confirmed that recombinants are rare (p<0.001) and non-persistent [18].

While adaptive mutation is known to provide RNA viruses with increased resistance to the immune system or therapeutics and to enhance their virulence [19–21], little is known as to what impact recombination may have in these same evolutionary mechanisms. Interestingly, gain of virulence may occur by acquisition of new genes or portions of genes during a recombination event, and represent a selective advantage. Gene duplication provide opportunities for species diversification, since genetic redundancy permits co-evolution of the duplicated genes. This in turn permits the acquisition of new biological functions or the modification of virulence. Although viral genomic duplications have been largely described in DNA viruses [22], they appear infrequent in RNA viruses due to biological constraints [23, 24], notably as concerns genome inflation. The few partial gene duplications reported in RNA viruses were located in untranslated regions of *Flaviviridae* [25, 26] or in short intragenic regions. Large insertions or duplications had never been reported in HCV before our recent identification of a duplicated NS5A-V3 region in genotype 1b [27].

The pleiotropic nature of NS5A in the HCV viral life cycle has sparked numerous analyses. The NS5A protein may be involved in resistance to interferon-based therapy, in hepatitis chronicity and in liver carcinogenesis [28]. NS5A also appears to be involved in viral particle assembly, giving it a critical role in the viral life cycle [29].

The recent use of anti-NS5A drugs, such as daclatasvir, in combination with other antiviral therapies is bringing even more attention to the protein [30, 31]. The V3 region (NS5A_2356-2379_) in the NS5A domain III was identified in 1991 [32]. Thereafter, our team and others went on to demonstrate [33] and confirm [34, 35] a correlation between the level of mutation in V3 and the response to interferon therapy.

In the present work, we analyzed the evolutionary history and dynamics of a previously reported set of HCV-1b strains bearing two tandemly repeated V3 regions in the NS5A gene (labelled R1 respectively and R2 as shown in the S1 Fig). In that previous work, we had identified a wide duplication event in the NS5A protein of a HCV-1b strain in a large prospective multicenter study in France including more than 800 patients (noted NS5A-dup hereafter) and demonstrated unprecedentedly that the duplication may be associated with a higher risk of liver complications [27]. Using phylogenomic analyses on two datasets: a direct sequence set (one sequence per patient) and a quasispecies (intra-host viral population) sequence set, we tested whether if the second V3 region arose via a duplication event or by another process like non-homologous recombination. Using molecular epidemiology tools, we compared mutant and wild-type strains within and between hosts.

We show here that the extra copy of the V3 region in NS5A did not arise by simple duplication. It is more likely that a double recombination event involving another HCV1-b is responsible for the doubling of the V3 region. Unfortunately, we could not identify a strain carrying a V3 region similar to the one found in the new type of NS5A protein. Finally, the relationships between a higher mutation rate and severity in hepatitis are discussed.

## Material and methods

### Samples

Two preexisting sample sets were used in the present study:

- a set of NS5A clonal quasispecies sequences taken from 10 HCV-1b infected patients included in the NAIF study. Nine of these patients had a wild NS5A gene and one a duplicated V3 gene. Two to four sequential samples were available for eight patients [34, 35].
- a set of direct NS5A sequences taken from the 27 patients who had been identified to be infected by a V3-duplicated HCV-1b in our previous studies [35].

We furthermore expanded our analyses using our French multicenter cohort: direct NS5A and E1 gene sequences were studied in 138 HCV strains (all the strains with the V3 duplication and a mean of ten strains taken from each center where a mutant strain had been identified; (S1 Table). A quasispecies study was also performed on 25 newly mutant strains identified with a sequential analysis in two strains (four and five sequential samples respectively). HCV sequences obtained in this study have been deposited in the DDBJ/EMBL/GenBank nucleotide sequence databases: accession numbers KU879336 to ku880537.

We also downloaded all the complete NS5A genes from all HCV genotypes available from the Los Alamos HCV Sequence Database [36].

### Ethical approval

As required by the Ethics Committee, an information letter was sent to all patients; a written or oral consent was not required as no additional serum sample was taken. Sera from consecutive patients newly diagnosed with chronic HCV-1b infection were obtained during the clinical work at the routine genotyping step in each participating virology laboratory (French academic centers: Angers; Avicenne; Besançon; Bordeaux; Brest; Clermont-Ferrand; Dijon; Grenoble; Limoges; Lyon; Nantes; Paris-Cochin; Paris-Pitié Salpétrière; Rennes; Saint-Etienne; Strasbourg; Toulouse; Tours). The research consisted in a non-interventional study, subsequently all data were analyzed anonymously. The study was approved by the Ethics Committee of the University Hospital of Brest (Avis CPP Ouest 6–15112006). The study was also registered with and approved by the national commission for information technology and civil liberties (CNIL, 907057–09032007).

### Experimental procedures

#### Direct NS5A sequences and NS5A quasispecies study

RNA extraction, entire NS5A gene reverse transcription, amplification, cloning and sequencing are explained in detail elsewhere [35]. Briefly, HCV RNA was extracted from 200 μL of each serum sample using the EasyMag automated extraction system (BioMérieux, Craponne, France). A full-length NS5A gene amplification was performed using outer primers E1 and E2 for the RT-PCR (1665 bp) and inner primers I3 and I41b (1344 bp) for the nested PCR. NS5A PCR products were subjected to direct sequencing using the Big Dye Terminator v3.1 Cycle Sequencing Kit (Applied Biosystems) on the automated ABI3130xl, with the primers used previously in the nested-PCR rounds. NS5A PCR products were also cloned using the p GEM-T Easy Vector, the T4 DNA ligase and JM109 High Efficiency Competent Cells (Promega) following the manufacturer’s instructions. Clones of interest, i.e., bearing the NS5A gene, were then sequenced as described above.

We sequenced a mean of 30 clones per sample and obtained 701 direct NS5A sequences and 1000 clonal NS5A sequences that were added to the previous sequence batches for analyses (37 direct NS5A sequences and 286 clonal NS5A sequences respectively).

#### E1 gene amplification and direct nucleotide sequencing

The E1 genomic segment (922 bp) was amplified with a two-step RT-PCR using an in-house protocol kindly provided by Professor C. Henquell. Briefly, an initial mixture containing 10μL of previously extracted HCV RNA, 0.4pM of random primers (Life Technologies) and 0.5mM of each dNTP (Life Technologies) was activated for 5 min at 65°C followed by 15 sec at 25°C. A second mixture containing 200U of SuperScript II Reverse Transcriptase (Invitrogen), 40U of RiboLock RNAse Inhibitor (Fermentas), 1X of reverse transcriptase buffer and 8m M of DTT for a 10μL final volume was added to the previous reaction tube. Reverse transcription was carried out for 10 min at 25°C and 50 min at 50°C. For PCR amplification, a 10μL aliquot of cDNA was then added to a 50μL final reaction volume. The reaction mixture contained 0.4μM of Env-S (5’-TGG GYA ARG TCA TCG ATA CC-3’) and Env-AS (5’-GGC 6GT 6CK RTT 6AT RTG CC-3’, 6 = inosine), forward and reverse primers respectively, 320μM of each dNTP, 1Xof polymerase buffer and 1.25U of *Taq* DNA polymerase (Qiagen). PCR was then performed with a DNA denaturation for 2 min at 95°C, followed by 45 cycles of denaturation (94°C for 15 sec), annealing (49°C for 50 sec) and elongation (72°C for 50 sec) and a final elongation for 5 min at 72°C. Samples bearing a V3 insertion and a mean of ten samples originating from each laboratory (9 to 17 samples) in which an insertion was identified were studied (138 samples).

E1 PCR products were subjected to direct sequencing using the Big Dye Terminators v3.1 Cycle Sequencing kit (Life Technologies) on the automated ABI3130xl, with forward and reverse primers used in the previous two-PCR round. The E1 nucleotide sequences were assembled into a consensus sequence then aligned to the HCVJ-1b prototype sequence (SeqScape software).

#### Sequences analysis

1286 sequences of the quasispecies NS5A gene were aligned according to their amino-acid sequence using the Muscle [37] alignment algorithm implemented in the MEGA v6.0.5 software [38]. Prior to any inference, the more likely substitution model was tested using jModelTest 2 [39]. The best model was found to be the HKY substitution model [40] and gamma heterogeneity and a proportion of invariant sites. This substitution model was employed when useful in all subsequent analyses. All genealogical and phylogenetic relationships between strains on NS5A and E1 genes were analyzed using the models implemented in the BEAST v1.8.2 software [41]. For each kind of analysis, we used empirical base frequencies, which prevent over-parameterization of the models.

Each analysis using BEASTv1.8 was run using both a strict and uncorrelated lognormal relaxed clock [42]. The number of iterations and sampling intervals were tuned for each analysis to obtain good ESS values (>200). Thus, the number of iterations varied from 2x10^7^ to 4x10^7^ and sampling intervals from 1,000 to 10,000. Estimated trees were analyzed using the TreeAnnotator software included in the BEAST package using a burn-in of 1000 and a posterior probability of 0.9. Several datasets were analyzed: intra-host analyses for NS5A carrying duplications or not, complete NS5A and E1 genes among wild and duplicated strains, NS5A V3 regions and NS5A without V3.

#### Intra-host dynamics

Clones of NS5A were sequenced in 55 quasispecies. First, we looked for occurrences of V3 region duplication to evaluate the homogeneity of NS5A types within a quasispecies. This was done because quasispecies composed only of NS5A-dup strains would favor the hypothesis of a clearly differentiated population over a recurrent mutational effect in the wild-type NS5A locus. Demographic dynamics of each quasispecies were inferred using the Bayesian Skyline plot [43] model implemented in BEAST. This model permits to plot the variation of effective size against evolutionary times given here in coalescent units (2 times the effective size *Ne*). Temporal samplings of a same quasispecies were grouped, decreasing the number of wild types from 18 to 9.

#### Differences in evolutionary rates between wild and NS5A-dup loci

To test for differences in the rates of evolution between NS5A-dup and wild loci, analyses were performed under the hierarchical phylogenetic model [44] using three strains with duplicated V3 regions and seven wild-type strains. HPM incorporates fixed effects to test for differences in evolutionary dynamics across host populations in a formal statistical framework employing stochastic search variable selection and model averaging [45]. Compared to the classical strategy of independent analyses of sequences from each patient, HPM provides a better estimation of evolutionary parameters such as nucleotide substitution rates. For the ten HCV strains, a temporal sampling was available, thus allowing for the estimation of the mutation rate of the locus [46] at the quasispecies level. According to hosts, the heterochronous sampling covers periods of several months (AG5sf) to 12 years (AG2) (see S1 Table). For patient AG2, we had serological and virological follow up around the renal graft period, which allowed us to demonstrate HCV seroconversion. Among the four temporal samples studied in the present work, we had the first positive HCV RNA just after the graft.

Statistical inferences of mutation rates and demography were performed using the extended Bayesian skyline plot (EBSP) model under the HPM framework. This model assumes population fluctuation, a common situation in viruses, especially in humans where treatments can strongly influence HCV demography.

#### Origins of NS5A-dup and the duplicated V3 region

The origins of the NS5A-dup were inferred from the analysis of their phylogenetic relationships with and divergence time from wild strains. We performed direct sequencing of the NS5A locus from 27 patients where the V3 duplication was found plus 70 patients carrying the wild type NS5A strains. We first used the complete sequences of the NS5A locus and thereafter the NS5A locus without the V3 region(s). For comparison purposes, the same analysis was performed for the E1 locus.

A second set of phylogenetic analyses were performed only on the V3 region. For the NS5A-dup locus, we compared the first copy of V3, labelled R1, with the second, labelled R2, treating them as different loci. We then tested the hypothesis of a separate origin of the extra copy of V3 in NS5A-dup, as opposed to a duplication.

The hypothesis of an origin of the supplementary V3 from outside of France was tested using 204 sequences of this locus in the Los Alamos database [36]. We performed all the analyses described in this section using the constant size coalescent model with random relaxed lognormal clock rate, which was found to better fit our data.

## Results

### Intra-host dynamics

A total of 55 quasispecies were studied. We found no patients carrying a mix of NS5A-dup and wild-type at any time, confirming the results of our recent clinical study [27] where patients were found to be infected by only one type of HCV strain, harboring NS5A-dup or not. However certain strains had two incomplete regions (RE1, LM1 and TR3), i.e., one complete V3 region and a piece of the second one, or in the case of TR3, the 5-prime part of the first region and the 3-prime part of the second.

**Fig 1:**
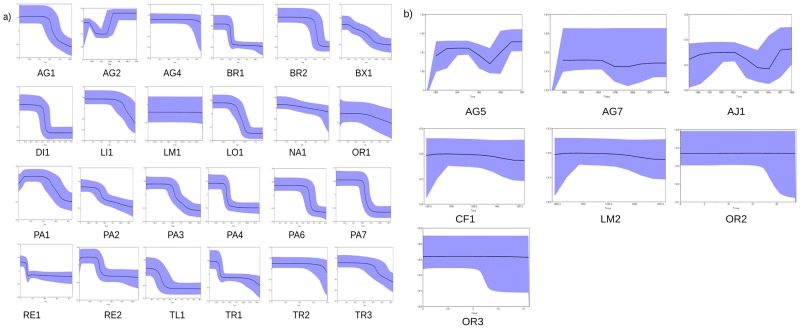
Intra-host demographic dynamics for NS5A-dup (A) and wild (B) strains of HCV1-b derived from Bayesian Skyline Plotting at the NS5A locus. When temporal sampling was available, time was scaled in years; otherwise time is given in coalescence time.

Except for strains AG3, AG6, BR3 and PA5 for which polymorphism was not sufficient to allow such inference, estimates of demographic dynamics using the Bayesian skyline model indicated that NS5A-dup strains had significantly greater demographic growth than wild ones (Mann-Whitney U, p = 0.001) (Fig 1). Bayesian skyline plots presented different shapes among the 26 NS5a-dup and the 9 wild strains. For every strain, demographic dynamics showed a plateau effect. Clear growth dynamics were nonetheless observed for NS5A-dup strains, whereas more constant population sizes were observed for wild types.

### NS5A-dup loci evolve faster than wild ones

**Fig 2:**
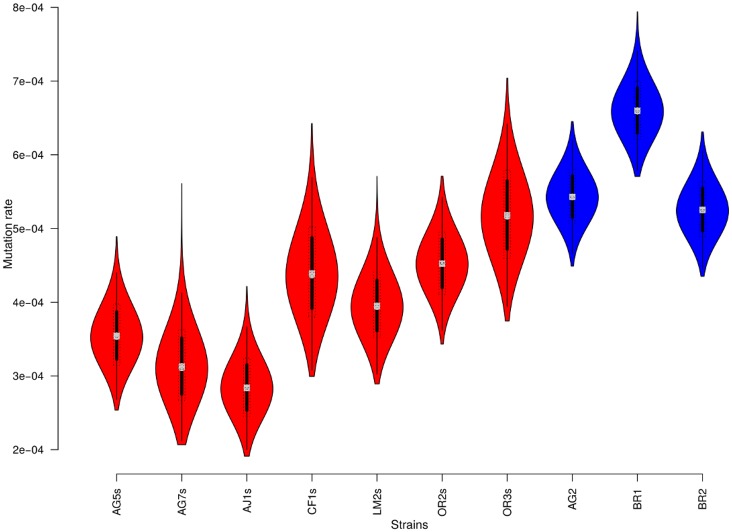
Posterior distributions of mean substitution rates of NS5a for each quasispecies of HCV1-b. Quasispecies carrying only one V3 domain (wild type) are represented in red; quasispecies carrying two V3 domains are represented in blue.

Hierarchical phylogenetic modelling (HPM) indicated that the NS5A-dup haplotypes had a greater mean mutation rate (5.74E-4±7.2E-5) than the wild-type haplotypes (3.94E-4±8.2E-5) (Mann-Whitney U, p<0.01) (Fig 2). Similarly, the tree heights for wild-type NS5A quasispecies were greater (42.71±5.4) than those of NS5A-dup quasispecies (33.05±2.4) (Mann-Whitney U, p<0.01).

### Origin of the extra V3 copy

**Fig 3:**
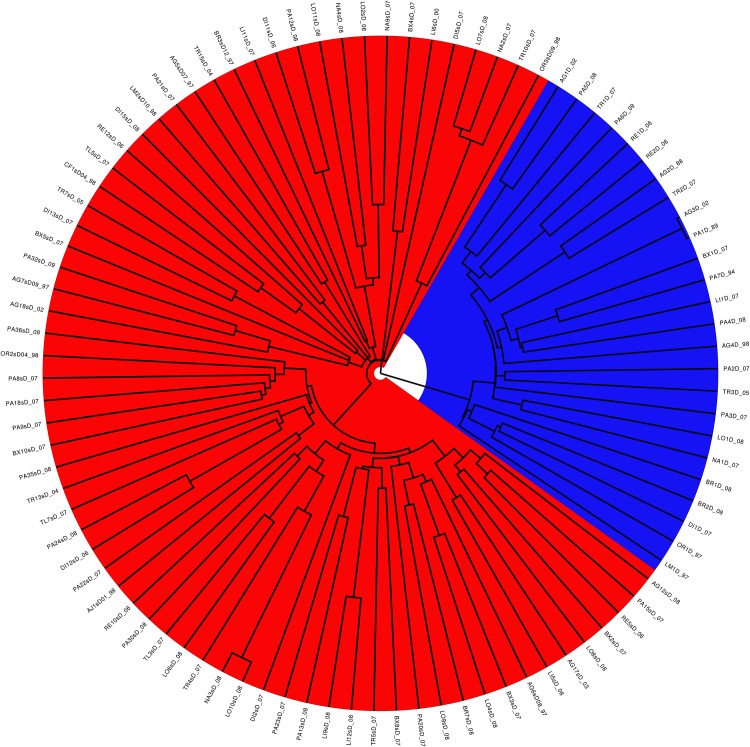
Divergce of the NS5A-dup strain from wild-type strains. Ninety percent posterior probability tree inferred from BEAST analysis under the coalescent constant size model with a random relaxed uncorrelated lognormal clock. NS5A-dup strains are indicated in blue. All strains carrying two V3-domains show common ancestry.

**Fig 4:**
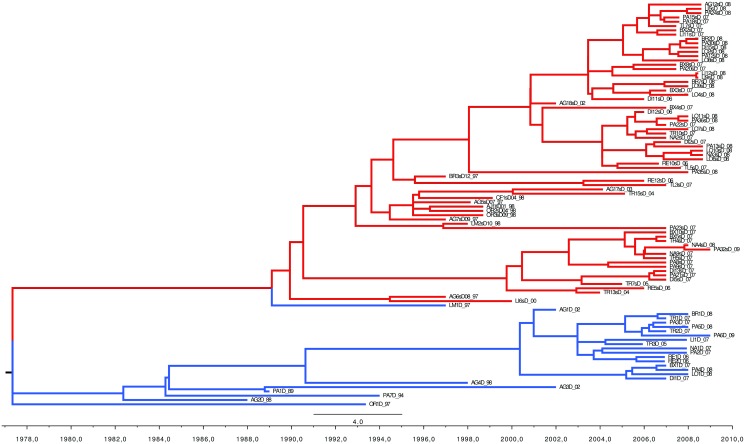
Divergence of NS5A-dup strains (in blue) from wild strains (in black). Ninety percent posterior probability tree inferred from BEAST analysis under the coalescent constant size model with a random relaxed uncorrelated lognormal clock. Age of nodes is mentioned on time-axis. The divergence between wild and duplicated type sequences is estimated to have occurred at the end of 70’s.

Fig 3 indicates that strains carrying NS5A-dup locus evolved separately from wild strains. To eliminate the differences induced by the presence of an extra V3 in NS5A-dup viruses, we performed the same analysis using sequences without the V3 region. This second analysis gave the same result (Fig 4), indicating that the clustering is genuinely due to a real divergence between strains. Strains carrying NS5A-dup appeared to have derived from the wild type in the last century. Finally, phylogeny of the E1 locus was congruent with that of the NS5A locus (S2 Fig).

**Fig 5:**
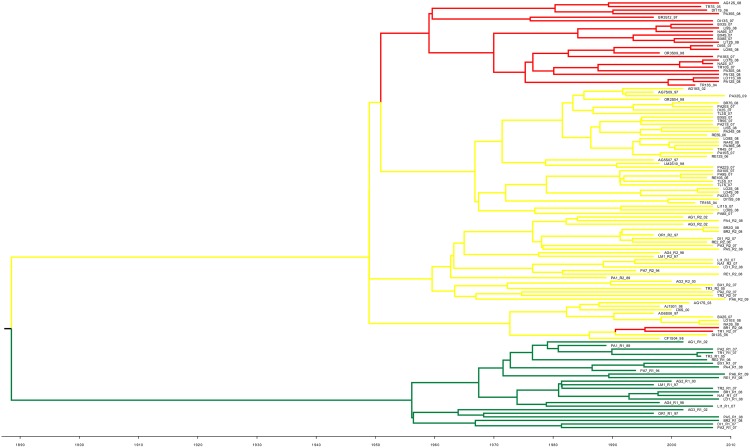
The origin of the first V3 region (R1, in green) in NS5A-dup strains differs from that of the second V3 region (R2, in red), which is more closely related to the wild-type region (in yellow). Ninety percent posterior probability tree inferred from BEAST analysis under the coalescent constant size model with a random relaxed uncorrelated lognormal clock. Time is on the x-axis and represents the divergence time between clades. Divergence between R1 and R2 is estimated to have occurred around 1920.

We also analyzed the V3 region alone. In NS5A-dup, the first and second copies were labelled R1 and R2 respectively. Fig 5 shows that R1 appears to be the most divergent, whereas R2 keep a closer relation with wild-type NS5A. This result is at odds with the hypothesis of a recent apparition of R1 by duplication of R2. Indeed, we hypothesized that R1 had another origin. This analysis of the V3 region alone also showed that the R1 cluster likely diverged from the others about a century ago. Although R2 copies clustered with wild-type regions, they formed a distinct clade, confirming an independent evolution.

**Fig 6:**
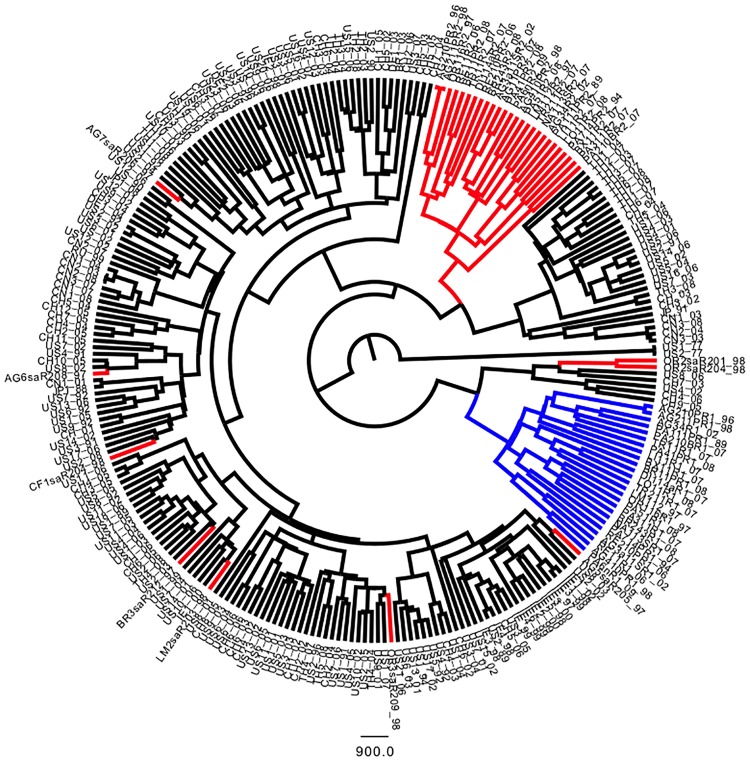
The origin of the R1 copy of the V3 region (in blue) differs from that of wild-type V3 regions (in black) at the world scale. R2 copies (in red) have a common origin with wild V3. Ninety percent posterior probability tree inferred from BEAST analysis under the coalescent constant size model with a random relaxed uncorrelated lognormal clock.

Finally, when performing the analysis with the Los Alamos HCV database, we found that R1 likely came from a HCV1-b genotype. However, no V3 region clearly clustered with the R1 copy (Fig 6). Thus, at this global scale, we confirmed the common origin of R2 copies with the wild-type V3 region.

## Discussion

Emergence of new pathogens is always of high interest, especially when it concerns human health. However, the biological mechanisms underlying emergence are poorly understood. In particular, the role of recombination in the creation of new viral populations is often underestimated as it needs co-infection events to occur. Even less well-described is the non-homologous recombination mechanism that can occur within or between genotypes of viruses. In the present work, we studied the evolutionary dynamics of new HCV1-b strains carrying two V3 regions instead of one at the gene encoding the NS5A protein. Using clone sequences of quasispecies, direct sequencing and heterochronous sampling, we showed that the new V3 region arose from non-homologous recombination events involving an unknown HCV1-b strain rather than by duplication. The population of NS5A-dup strains then probably derived from wild-type strains about a century ago. Finally, the increased evolutionary capacity of the NS5A protein with two V3 regions may influence the severity of hepatitis C in humans, as our recent clinical work suggested [27, 47].

### Origins of the NS5A-dup strains

Our phylogenetics analyses, whatever the dataset used (on NS5A including or excluding the V3 region or on the E1 gene), all reached the same conclusion that the NS5A-dup strains diverged about a century ago from wild-type HCV1-b viruses. Also, the direct sequences of wild-type quasispecies gave greater tree heights than those of NS5A-dup quasispecies, confirming that the latter appeared more recently. Thus, NS5A-dup HCV1-b strains appear to constitute a real emergence of a new HCV1-b family. The clear-cut clustering between wild-type and NS5A-dup strains suggests a reproductive isolation between these two families. Free mixing between wild-type and NS5A-dup strains would have left traces of recombinations in the NS5A gene sequences. The likelihood of such recombination events here suggests multiple infections from different donors, allowing the mixing of several populations of strains within a patient. Our dataset did not show any evidence of such mixing of populations within any of our patients. Consequently, there is little chance for recombination between the two families of HCV1-b to occur in a short period of several decades. In the absence of recombination events between these strains, the question of reproductive isolation within HCV1-b is still pending. Is recombination lacking because of its rarity in such viruses [18, 48, 49] or is it occurring but deleterious because of a damaging phenotypic effect such as incompatibilities [50, 51]? Toward this, experimental recombination assays *in vitro* would help to shed light on the reality of reproductive isolation.

Also particularly interesting were the phylogenetics of the V3 copies themselves. We found that the first V3 copy did not cluster with the second one. In the case of duplication, a common origin for the two copies would be expected, but our results were at odds with that logic. Indeed, the R1 copy of V3 appeared to come from another population of HCV1-b, as illustrated by Figs 5 and 6, whereas the R2 copy appeared to derive from the V3 region observed in the wild-type NS5A genes. The comparison with NS5A haplotypes from the Los Alamos database did not permit to clearly identify a potential ancestor for the R1 copy, but comparisons with other genotypes of HCV clearly showed that it did cluster with HCV1-b genotypes.

The mechanism by which the R1 was added to the 5-prime side of the original V3 region remains unclear. We hypothesize here that this extra copy may have been introduced by non-homologous recombination between two divergent HCV1-b strains. Non-homologous recombination in HCV was recently described by Scheel *et al*. [48], who demonstrated the potential for non-homologous recombination in RNA-viruses using cell cultures. To our knowledge, our present work is the first to report a viable non-homologous recombination in a RNA virus that led to the emergence of a new family of HCV1-b about a century ago. Homologous recombination, although rare in certain viruses, has been recognized to involve positive epistasis and produce recombinants with increased fitness [52]. In contrast, non-homologous recombination is often deleterious and likely to produce non-viable genotypes. In their experiment, Scheel *et al*. observed a progressive deletion of the largest extra copies of genomes (1065 nts) introduced by non-homologous recombination, with a rapid evolution toward more fit homologous recombinants with smaller 186 nts duplicated regions following passaging in cell culture [48]. We also observed the occurrence of incomplete V3 regions in three direct sequences of NS5A-dup strains and also in their respective entire quasispecies. Such partial deletions may suggest that the duplicated V3 region is slightly deleterious. Scheel *et al*. [48], in their *in vitro* experiment, showed that the elimination or excision of extra copies occurs in only a few generations. However, recombinants with smaller duplicated sequences appeared remarkably stable, similarly to our observations in the natural HCV recombinants. The same results were also obtained recently by Holmblat *et al*., who reported that *in vitro* non-homologous recombination between defective poliovirus and coxsackievirus led to durable duplications with no alteration of neurovirulence in mice [53]. They also showed that non-homologous recombinants with large inserts (>70 codons) submitted to successive homologous recombination evolve to a more stable state, and moreover confirmed that recombinants with small duplicated regions were stable. They concluded that these recombinations may be viewed as a model of genetic plasticity for enteroviruses. We did not observe quick elimination of the extra V3 region in our study, quite the contrary they seemed to have persisted for about a century. In contrast to the work of Scheel *et al*. [48], the V3 duplication reported here has experienced “real life” in human organisms, and more recently, faced clinical treatments.

We suggest that far from being deleterious, the supplementary V3 region may provide an epidemiological advantage for HCV1-b. This hypothesis is supported by the observation of better demographic dynamics (Fig 1) and intra-host mutation rates (Fig 2) for NS5A-dup. Other examples of advantageous duplications in viruses can be found in the literature. For example, a duplication in the P1 gene of *Potyviridae*, a family of plant viruses, was reported to be the consequence of a former genomic recombination and to largely contribute to their adaptation to a wide range of host species [54]. In another report on the bovine viral diarrhea virus, another *Flaviviridae* belonging to the Pestivirus genus, a duplicated NS3 gene was associated with increased disease severity [55]. In the same way, our results showed that the introduction of an extra copy of V3 did not alter the fitness of HCV strains, and the results of our recent clinical work suggest that such strains may have more negative effects on the patients than their wild counterparts [27, 47].

### Implications for viral emergence

The evolutionary rate of the NS5A-dup gene was greater than that of the wild-type locus. HPM allowed us to obtain a good estimate of the mutation rate for each quasispecies. The capacity of HPM to compare intra-host dynamics has been demonstrated in HIV [45]. Its results are more accurate than those obtained from working on each quasispecies independently.

The difference in evolutionary rates between NS5A-dup and wild loci had no consequence on the demographic dynamics of the different families as illustrated by the demographic curves estimated under the Bayesian Skyline Model in Fig 1. Also, our clinical study did not observe differences in viral load levels between patients infected by the NS5A-dup strain and those infected by wild strains [27].

Thus, although carrying two V3 regions may not confer a net advantage in the intra-host population growth rate, it also does not appear to have any deleterious effects. Moreover, we have detected the strain in a number of patients. Its persistence in patients infected at different times again argues in favor of the absence of a deleterious effect of the extra V3 copy. In other words, the acquisition of a supplementary V3 region should be considered as selectively neutral at least, if not positive.

In the present work, we showed for the first time the impact of non-homologous recombination on the emergence of a new family of HCV1-b strains. Recombination, although rare in viruses, could nonetheless play an important role in the potential of emergence.

However, the recombination rate in HCV may be underestimated, probably due to clinical diagnostic methods, which usually focus on a single small region rather than large genomic portions. Here we report a non-homologous recombination, an event thought to be rarer than homologous recombination. Our finding may have important consequences on HCV epidemiology. Indeed, as we demonstrated, the NS5a-dup strains may form a distinct clade, supposing a recent reproductive isolation from wild strains. This new population should have its own dynamics, and thus considering HCV1-b as a single population entity for epidemiology could be a source of bias. Toward the question as to whether NS5A-dup represents a new emergent HCV-1b cluster, it would be of use to question the occurrence of such non-homologous recombination events in other viruses for which recombination is assumed to be more likely.

## Supporting information

S1 Fig Alignments of two batches of direct sequences taken from wild type NS5A strains and NS5A-dup mutants strains respectively. The first V3 domain, labelled V3-R1 (light grey square), is located between positions 2353 and 2383, the second V3 domain, labelled V3-R2 (dark grey square), is tandemly located thereafter out to position 2414. This second domain is aligned with the unique V3 domain from the wild-type NS5A strains (light brown square). Dashes (-) in the NS5A-dup sequences indicate shorter deletions in the last 6 sequences.
(TIF)

S2 Fig Phylogeny of the E1 locus. NS5A-dup strains (in blue) are divergent from wild ones (in red). Ninety percent posterior probability tree inferred from BEAST analysis under the coalescent constant size model with random relaxed uncorrelated lognormal clock.
(TIF)

S1 Table Description of the strains of HCV1-b. For each strain the locality and date of sampling are given. Sequences of NS5A harboring two V3 regions are indicated in bold and italics, the others are wild type. Direct sequences are given in S1A Table and strains studied for quasispecies analysis are given in S1B Table.
(DOCX)
