# Supplementary figures and images for: Natural non-homologous recombination led to the emergence of a duplicated V3-NS5A region in HCV-1b strains associated with hepatocellular carcinoma
Source: PLoS One. 2017 Apr 10;12(4):e0174651. doi: 10.1371/journal.pone.0174651 (PMC5386276; doi:10.1371/journal.pone.0174651)

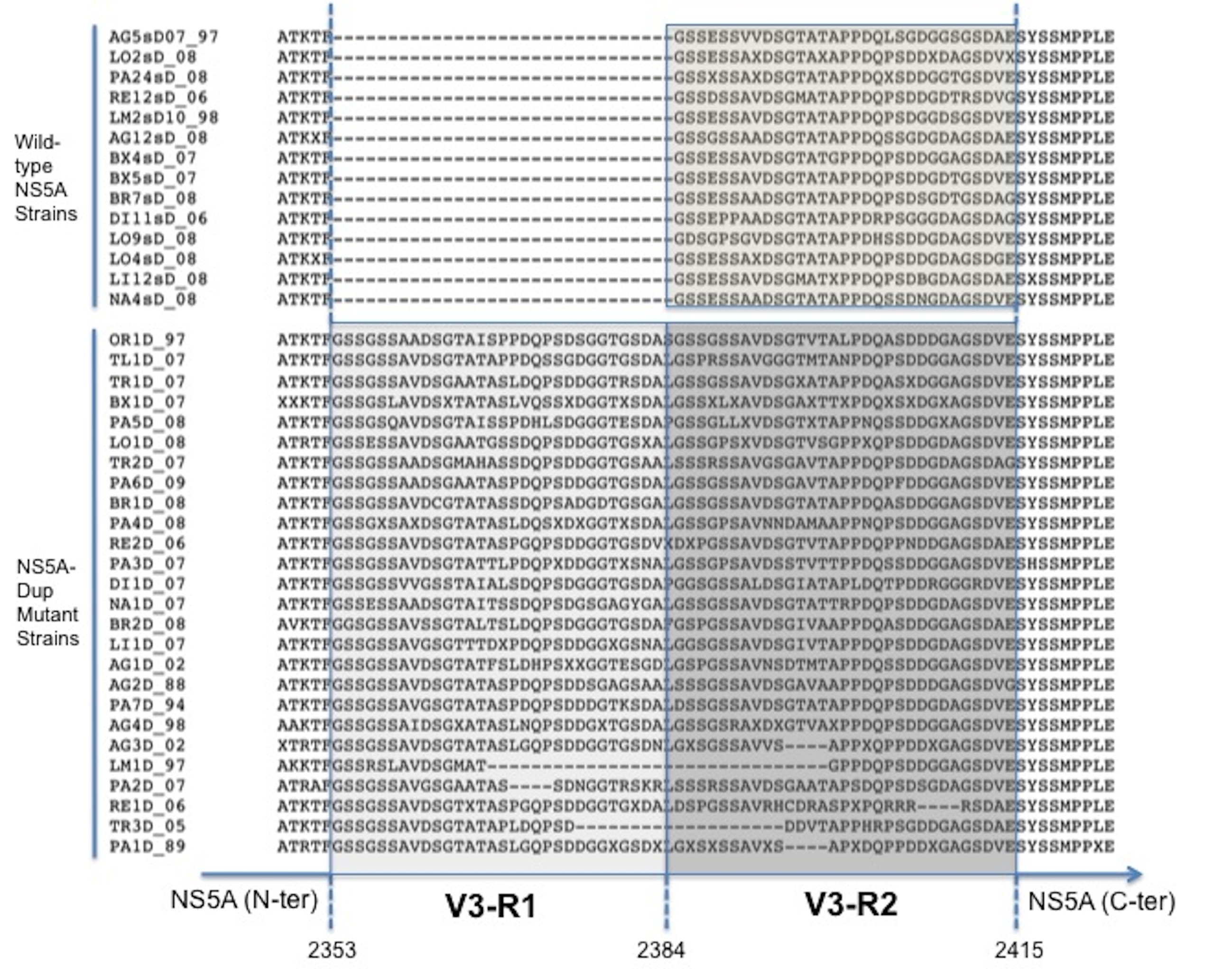

Supplement: S1 Fig — The first V3 domain, labelled V3-R1 (light grey square), is located between positions 2353 and 2383, the second V3 domain, labelled V3-R2 (dark grey square), is tandemly located thereafter out to position 2414. This second domain is aligned with the unique V3 domain from the wild-type NS5A strains (light brown square). Dashes (-) in the NS5A-dup sequences indicate shorter deletions in the last 6 sequences. (TIF) [file pone.0174651.s001.tif]

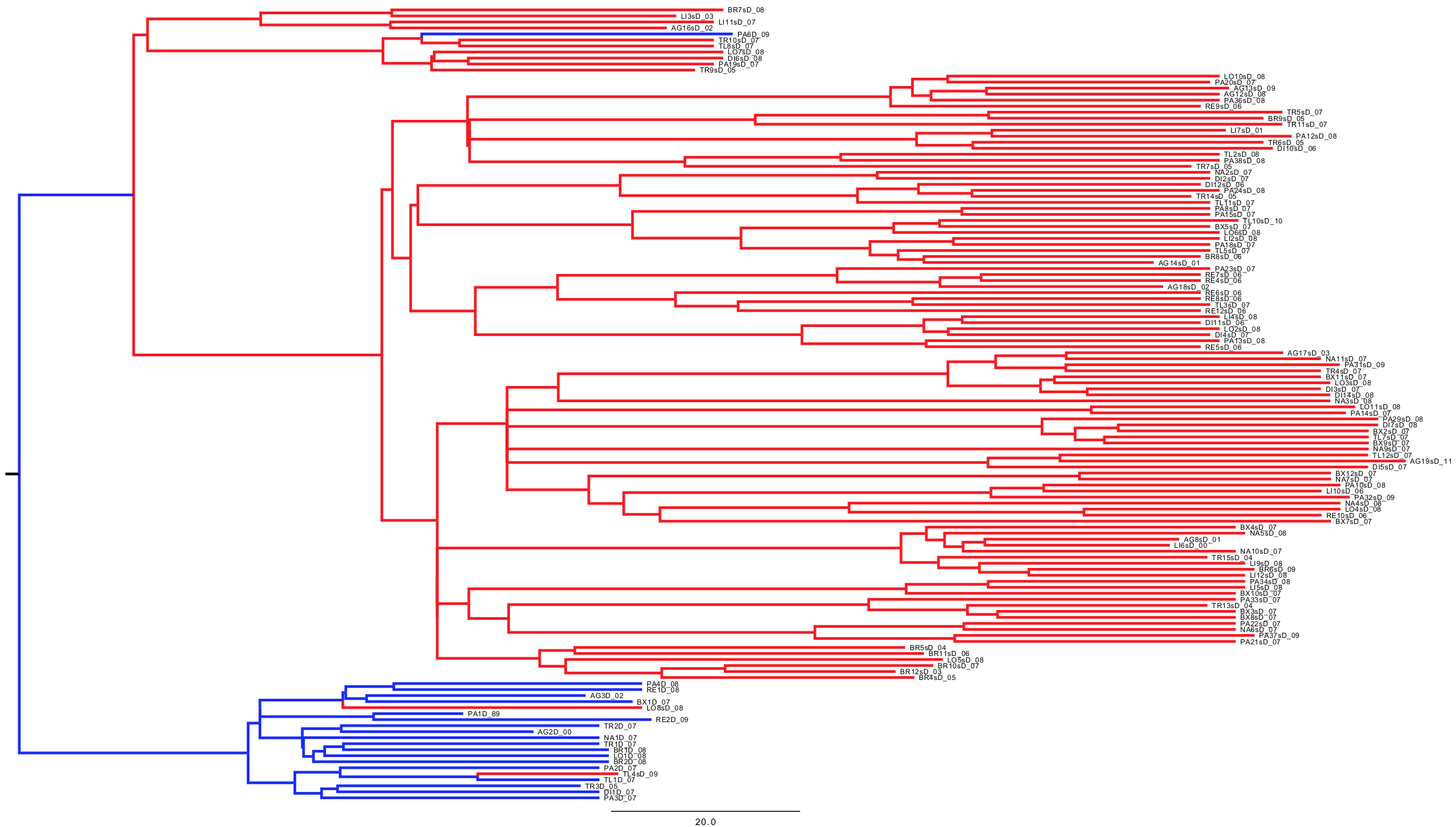

Supplement: S2 Fig — Ninety percent posterior probability tree inferred from BEAST analysis under the coalescent constant size model with random relaxed uncorrelated lognormal clock. (TIF) [file pone.0174651.s002.tif]
