# Supplementary material for: Natural non-homologous recombination led to the emergence of a duplicated V3-NS5A region in HCV-1b strains associated with hepatocellular carcinoma
Source: PLoS One. 2017 Apr 10;12(4):e0174651. doi: 10.1371/journal.pone.0174651 (PMC5386276; doi:10.1371/journal.pone.0174651)
Supplement: S1 Table — For each strain the locality and date of sampling are given. Sequences of NS5A harboring two V3 regions are indicated in bold and italics, the others are wild type. Direct sequences are given in S1A Table and strains studied for quasispecies analysis are given in S1B Table. (DOCX) [file pone.0174651.s003.docx]

**S1 Table 1. Description of the strains of HCV1-b.** For each strain the locality and date of sampling are given. Sequences of NS5A harbouring two V3 domains are indicated in bold and italic, the others belong to wild type. Direct sequences are given in Table 1A and strains studied for quasispecies analysis are given in Table 1B with the number of clonal sequences obtained for each sample (n).

Table 1A

| **Locality** | **Code** | **Date** |
| --- | --- | --- |
| **Angers** | AG8s | 23/01/2001 |
|  | AG9s | 01/09/2004 |
|  | AG10s | 04/04/2006 |
|  | AG11s | 01/02/1999 |
|  | AG12s | 16/06/2008 |
|  | AG12s | 03/02/2009 |
|  | AG14s | 01/01/2001 |
|  | AG15s | 01/05/2004 |
|  | AG16s | 12/03/2007 |
|  | AG17s | 01/08/2003 |
|  | AG18s | 01/11/2002 |
|  | AG19s | 23/11/2011 |
| **Bordeaux** | BX2s | 30/01/2007 |
|  | BX3s | 23/05/2007 |
|  | BX4s | 11/06/2007 |
|  | BX5s | 19/09/2007 |
|  | BX6s | 05/03/2007 |
|  | BX7s | 09/08/2007 |
|  | BX8s | 17/07/2007 |
|  | BX9s | 23/05/2007 |
|  | BX10s | 15/10/2007 |
|  | BX11s | 05/03/2007 |
|  | BX12s | 23/05/2007 |
| **Brest** | BR4s | 30/06/2005 |
|  | BR5s | 26/01/2004 |
|  | BR6s | 03/02/2009 |
|  | BR7s | 24/09/2008 |
|  | BR8s | 18/01/2006 |
|  | BR9s | 15/02/2005 |
|  | BR10s | 26/04/2007 |
|  | BR11s | 31/01/2006 |
|  | BR12s | 13/03/2003 |
| **Dijon** | DI2s | 10/10/2007 |
|  | DI3s | 04/07/2007 |
|  | DI4s | 19/01/2007 |
|  | DI5s | 21/05/2007 |
|  | DI6s | 06/03/2008 |
|  | DI7s | 08/01/2008 |
|  | DI8s | 03/09/2007 |
|  | DI9s | 26/09/2007 |
|  | DI10s | 09/11/2006 |
|  | DI11s | 21/11/2006 |
|  | DI12s | 15/12/2006 |
|  | DI13s | 04/07/2007 |
|  | DI14s | 04/03/2008 |
|  | DI15s | 17/01/2008 |
| **Limoges** | LI2s | 09/01/2008 |
|  | LI3s | 02/09/2003 |
|  | LI4s | 05/05/2000 |
|  | LI5s | 07/01/2008 |
|  | LI6s | 04/05/2000 |
|  | LI7s | 05/02/2001 |
|  | LI8s | 12/12/2003 |
|  | LI9s | 31/03/2008 |
|  | LI10s | 20/09/2006 |
|  | LI11s | 03/09/2007 |
|  | LI12s | 18/03/2008 |
| **Lyon** | LO2s | 08/07/2008 |
|  | LO3s | 06/06/2008 |
|  | LO4s | 19/08/2008 |
|  | LO5s | 04/04/2008 |
|  | LO6s | 26/08/2008 |
|  | LO7s | 03/06/2008 |
|  | LO8s | 08/07/2008 |
|  | LO9s | 02/07/2008 |
|  | LO10s | 01/09/2008 |
|  | LO11s | 14/04/2008 |
| Nantes | NA2s | 06/12/2007 |
|  | NA3s | 07/01/2008 |
|  | NA4s | 11/02/2008 |
|  | NA5s | 22/01/2008 |
|  | NA6s | 23/10/2007 |
|  | NA7s | 06/04/2007 |
|  | NA8s | 01/10/2007 |
|  | NA9s | 12/07/2007 |
|  | NA10s | 13/12/2007 |
|  | NA11s | 20/11/2007 |
| Paris | PA8s | 11/01/2007 |
|  | PA9s | 05/03/2007 |
|  | PA10s | 07/01/2008 |
|  | PA11s | 22/02/2008 |
|  | PA12s | 09/01/2008 |
|  | PA13s | 26/03/2008 |
|  | PA14s | 31/07/2007 |
|  | PA15s | 17/01/2007 |
|  | PA16s | 26/01/2007 |
|  | PA17s | 27/03/2007 |
|  | PA18s | 22/08/2007 |
|  | PA19s | 13/11/2007 |
|  | PA20s | 15/11/2007 |
|  | PA21s | 03/12/2007 |
|  | PA22s | 26/04/2007 |
|  | PA23s | 09/05/2007 |
|  | PA24s | 25/03/2008 |
|  | PA29s | 13/06/2008 |
|  | PA30s | 04/04/2008 |
|  | PA31s | 11/03/2009 |
|  | PA32s | 11/06/2009 |
|  | PA33s | 20/12/2007 |
|  | PA34s | 15/05/2008 |
|  | PA35s | 02/04/2008 |
|  | PA36s | 29/02/2008 |
|  | PA37s | 18/05/2009 |
|  | PA38s | 18/09/2008 |
| Rennes | RE3s | 18/09/2006 |
|  | RE4s | 03/11/2006 |
|  | RE5s | 23/02/2006 |
|  | RE6s | 16/08/2006 |
|  | RE7s | 23/10/2006 |
|  | RE8s | 26/09/2006 |
|  | RE9s | 31/05/2006 |
|  | RE10s | 27/06/2006 |
|  | RE11s | 28/02/2006 |
|  | RE12s | 06/12/2006 |
| Toulouse | TL2s | 11/02/2008 |
|  | TL3s | 02/02/2007 |
|  | TL4s | 06/07/2009 |
|  | TL5s | 23/04/2007 |
|  | TL6s | 28/05/2009 |
|  | TL7s | 27/11/2007 |
|  | TL8s | 12/06/2007 |
|  | TL9s | 22/01/2009 |
|  | TL10s | 11/01/2010 |
|  | TL11s | 06/09/2007 |
|  | TL12s | 25/06/2007 |
|  | TL13s | 17/01/2007 |
|  | TL14s | 05/01/2007 |
| Tours | TR4s | 11/04/2007 |
|  | TR5s | 21/06/2007 |
|  | TR6s | 23/05/2005 |
|  | TR7s | 04/11/2005 |
|  | TR8s | 22/10/2007 |
|  | TR9s | 11/05/2005 |
|  | TR10s | 12/01/2007 |
|  | TR11s | 26/02/2007 |
|  | TR12s | 01/01/2005 |
|  | TR13s | 31/03/2004 |
|  | TR14s | 21/01/2005 |
|  | TR15s | 18/05/2004 |

Table 1B

| **Locality** | **Code** | **Date** | **n** |
| --- | --- | --- | --- |
| **Ajaccio** | AJ1s | 24/09/1997 | 16 |
|  |  | 25/01/1998 | 23 |
| **Angers** | ***AG1*** | ***01/08/2002*** | ***26*** |
|  | ***AG2*** | ***18/02/2000*** | ***32*** |
|  |  | ***07/10/1998*** | ***24*** |
|  |  | ***16/07/1996*** | ***26*** |
|  |  | ***12/04/1988*** | ***30*** |
|  | ***AG3*** | ***01/01/2002*** | ***32*** |
|  | ***AG4*** | ***29/06/1998*** | ***28*** |
|  | AG5s | 09/05/1997 | 15 |
|  |  | 29/07/1997 | 17 |
|  | AG6s | 29/09/1997 | 15 |
|  | AG7s | 16/06/1997 | 17 |
|  |  | 16/09/1997 | 6 |
| **Bordeaux** | ***BX1*** | ***25/06/2007*** | ***29*** |
| **Brest** | ***BR1*** | ***11/07/2008*** | ***35*** |
|  |  | ***11/06/2007*** | ***38*** |
|  |  | ***20/03/2006*** | ***37*** |
|  |  | ***14/03/2005*** | ***43*** |
|  |  | ***06/01/2004*** | ***37*** |
|  | ***BR2*** | ***03/03/2008*** | ***29*** |
|  |  | ***03/04/2006*** | ***20*** |
|  |  | ***04/03/2004*** | ***13*** |
|  |  | ***15/07/2002*** | ***27*** |
|  | BR3s | 28/01/1998 | 13 |
| **Clermont-Ferrand** | CF1s | 17/09/1997 | 17 |
|  |  | 23/01/1998 | 10 |
|  |  | 24/04/1998 | 4 |
| **Dijon** | ***DI1*** | ***17/01/2007*** | ***30*** |
| **Le Mans** | ***LM1*** | ***14/03/1997*** | ***7*** |
|  | LM2s | 13/03/1998 | 17 |
|  |  | 24/07/1998 | 12 |
|  |  | 14/10/1998 | 11 |
| **Limoges** | ***LI1*** | ***17/01/2007*** | ***30*** |
| **Lyon** | ***LO1*** | ***13/05/2008*** | ***33*** |
| **Nantes** | ***NA1*** | ***15/05/2007*** | ***32*** |
| **Orléans** | ***OR1*** | ***10/12/1997*** | ***8*** |
|  | OR2s | 28/10/1997 | 14 |
|  |  | 21/01/1998 | 11 |
|  |  | 15/04/1998 | 12 |
|  | OR3s | 07/07/1998 | 15 |
|  |  | 28/09/1998 | 15 |
| **Paris** | ***PA1*** | ***28/11/1989*** | ***29*** |
|  | ***PA2*** | ***02/04/2007*** | ***31*** |
|  | ***PA3*** | ***28/12/2007*** | ***31*** |
|  | ***PA4*** | ***07/03/2008*** | ***29*** |
|  | ***PA5*** | ***05/03/2008*** | ***31*** |
|  | ***PA6*** | ***25/05/2009*** | ***27*** |
|  | ***PA7*** | ***19/09/1994*** | ***32*** |
| **Rennes** | ***RE1*** | ***14/10/2008*** | ***39*** |
|  | ***RE2*** | ***17/12/2009*** | ***21*** |
| **Toulouse** | ***TL1*** | ***12/03/2007*** | ***31*** |
| **Tours** | ***TR1*** | ***19/02/2007*** | ***33*** |
|  | ***TR2*** | ***30/10/2007*** | ***29*** |
|  | ***TR3*** | ***01/01/2005*** | ***27*** |
